# Editorial: New advances in identification and quantification of foodborne pathogens, volume II

**DOI:** 10.3389/fmicb.2023.1205923

**Published:** 2023-05-12

**Authors:** Dario De Medici, Nigel Cook, David Rodriguez-Lazaro

**Affiliations:** ^1^Department of Food Safety, Nutrition and Veterinary Public Health, Istituto Superiore di Sanità, Rome, Italy; ^2^Jorvik Food and Environmental Virology Ltd., York, United Kingdom; ^3^Microbiology Division, Faculty of Sciences, University of Burgos, Burgos, Spain; ^4^Centre for Emerging Pathogens and Global Health, University of Burgos, Burgos, Spain

**Keywords:** food safety, molecular methods detection, foodborne pathogen detection, biosensors, Raman micro-spectroscopy (RMS), image analysis, loop mediated isothermal amplification (LAMP), *Vibrio parahaemolyticus*

After the extensive contributions to the first volume of the Research Topic “*New advances in identification and quantification of foodborne pathogens*,” in which 12 original articles highlighted the major benefits and weaknesses of rapid methods for the detection of microbial pathogens in foods, five original articles continue the discussion within this second volume.

All the articles, in both volumes 1 and 2, affirm that rapid and precise detection (and/or quantification) of foodborne pathogens, used appropriately along the entire food supply chain, represents a powerful tool for the control and prevention of outbreaks of foodborne disease. Rapid methods, such as the various molecular techniques and biosensor-based methods which have been developed for food control, need to be better integrated as part of food safety management, because, as has been repeatedly discussed, they are more rapid, sensitive, accurate, and cost-affordable than culture-based methods.

A comprehensive review, with the aim to thoroughly discuss the recent advances, applications, and limitations of portable and rapid biosensors proposed for routine analysis of foodborne pathogens, is presented by Quintela et al. The authors emphasize that biosensors inherently display the characteristics that the World Health Organization (WHO) has established with the acronym ASSURED (Affordable, Sensitive, Specific, User-friendly, Rapid and Robust, Equipment-free, and Delivered), particularly as the rapid tests can be usable in areas and production settings with limited resources. The authors stress the ability of biosensor-based methods to detect and measure specific pathogens even at low concentrations, requiring only small volumes of samples for analysis, which makes their deployment both efficient and convenient, especially in areas where many samples need to be tested immediately. Biosensors have been demonstrated to be very useful for rapid screening for the presence of foodborne pathogens in various matrices, within minutes or hours, with or without pre-treatment steps.

Zhang et al. propose the application of confocal Raman micro-spectroscopy as a fast and easy-to-use method for the efficient detection and identification of the foodborne pathogenic bacteria responsible for the majority of serious foodborne illnesses. The differences in composition of various bacterial pathogens at the molecular level, were detected by the Raman spectrum, which is typical of each microorganism's biochemical composition. These vibrational spectra were able to discriminate between six different foodborne pathogenic bacteria as *Salmonella* Typhimurium, *Shigella flexneri, Listeria monocytogenes, Vibrio cholerae, Staphylococcus aureus*, and *Clostridium botulinum*. The proposed method was demonstrated to be a powerful analytical technique for the rapid characterization and detection of bacteria without external labels or tedious preparation.

The inability to discriminate between viable and dead cells (or infectious and non-infectious virus particles) remains one of the main weaknesses of the majority of the rapid methods (De Medici et al., 2021). Detection of live/viable bacteria, eliminating false-positive results caused by detection of dead cells, represents a critical factor and the main obstacle for complete acceptance of molecular based methods in food safety management systems by producers and regulatory agencies.

A novel approach for detecting bacteria based on the imaging-based quantification of phage amplification, improving the specificity of discriminating live vs. dead bacteria, is proposed by Wisuthiphaet et al. This study uses the natural ability of lytic bacteriophages to rapidly amplify their genetic material and generate progeny phages upon infecting the host live bacterium. The amplification of the bacteriophages was detected by a nucleic acid staining dye (SYBR-Green), a conventional fluorescence microscope, and a quantitative image analysis. Sensitivity and assay time for imaging-based quantification of phage amplification were compared with RT-PCR and standard plaque-forming assay. The effectiveness of the method was evaluated by detecting the presence of *E. coli* in two food system models, i.e., coconut water and simulated spinach wash water. The imaging method was demonstrated to be able to detect 10 CFU/ml of *E. coli* in coconut water and spinach wash water after, respectively, 2 and 1 h.

Busch et al. proposed an interesting two-step LAMP assay for the specific detection of *Bacillus cereus*-group (*B. cereus* group) and the potentially non-hemolytic enterotoxin (NHE)-producing cells. The paper proposed an interesting solution for rapid screening of colonies isolated from foodstuffs within 24 h. Specific sequences were used to determine the representatives of the *B. cereus*-group, and cells capable of producing enterotoxins. The suitability of the LAMP assay for detecting the *B. cereus* group and the toxin gene *nheB* from the food matrix was tested by means of spiked samples. The specificity of the developed assay was 100% for *B. cereus*-group isolates and 93.7% for the detection of *nhe*B toxin gene. In addition, 80 raw meat products such as minced meat, and cooked samples such as meatballs and 20 different vegetarian substitute products, were tested for the presence of presumptive *B. cereus* in accordance with DIN EN ISO standard 21871 and extracted DNA was checked using both LAMP assays and both real-time PCR assays. The results showed that in those food samples, the relevant contamination levels could be reliably detected by two-step LAMP within 24 h. It is noteable that the culture-based detection required more work and time while the real-time PCR used for comparison did not show such reliable detection rates.

In the last article of this Research Topic, He et al. investigated a protein secreted by *V. parahaemolyticus*, which is associated with an integrative and conjugative element (ICE). Based on proteomics analysis this protein was identified as dihydrolipoamide dehydrogenase. The paper may provide new data relevant to understand better the virulence or stress adaptation of *Vibrio parahemolyticus*. However, the biological functions associated with the virulence of *V. parahaemolyticus* remain unknown. The findings from this study should enhance the understanding of mobile genetic elements, and the remaining unresolved questions should facilitate further studies that focus on elucidating the pathogenesis of *V. parahaemolyticus* and detecting diagnostic markers.

## Author contributions

All authors listed have made a substantial, direct, and intellectual contribution to the work and approved it for publication.
